# A case of thoracoscopic medial basal segmentectomy

**DOI:** 10.1016/j.ijscr.2018.12.004

**Published:** 2019-01-11

**Authors:** Naoya Kawakita, Hiroaki Toba, Shoji Sakiyama, Mitsuhiro Tsuboi, Hiromitsu Takizawa, Akira Tangoku

**Affiliations:** a Department of Thoracic and Endocrine Surgery and Oncology, Institute of Health Biosciences, The University of Tokushima Graduate School, Tokushima, Japan; b Department of Thoracic Surgery, Kochi National Hospital, Kochi, Japan

**Keywords:** CT, computed tomography, IPV, inferior pulmonary vein, ICS, intercostal space, Segmentectomy, Metastasectomy, Thoracoscopy

## Abstract

**Introduction:**

Isolated resection of the medial basal segment (S7) is uncommon because of its small volume, and S7 segmentectomy is considered to be difficult due to anatomical variation. We report a case of successful thoracoscopic S7 segmentectomy.

**Presentation of case:**

A 56-year-old man was referred to our hospital with suspected pulmonary metastasis of rectal cancer. A 6-mm nodule was detected in S7. A7 and B7 branched from the basal segmental artery and bronchus, respectively, to run ventral to the inferior pulmonary vein. This made it possible to isolate A7 and B7 by an approach via the interlobar fissure.

In addition, V7a and V7b were easily isolated from inferior pulmonary vein. The intersegmental plane was indicated by V7b and was transected along a demarcation line identified by using selective oxygenation via B7.

**Discussion:**

B7 most commonly branches from the basal bronchus and A7 from the basal artery to run ventral to the inferior pulmonary vein. With this anatomical type, when the surgeon approaches via the interlobar fissure during surgery, A7 is identified first, B7 is seen behind A7, and the IPV is posterior to B7. Since the intersegmental plane is located ventral to the IPV, segmentectomy can be completed via the interlobar fissure approach.

**Conclusion:**

In patients with this pattern of pulmonary artery and bronchial anatomy, isolated S7 segmentectomy is a feasible treatment option.

## Highlights

• Medial basal segmentectomy is uncommon procedure.
• It is difficult to perform the procedure because of anatomical variations.
• This is a rare report of thoracoscopic medial basal segmentectomy.

## 1 Introduction

The right medial basal segment (S7) has the smallest volume of all lung segments [1] and shows anatomical variation, so medial basal segmentectomy is thought to be difficult and very few cases have been reported [2,3]. Here we report a patient with a metastatic lung tumor in whom medial basal segmentectomy was performed by video-assisted thoracoscopic surgery. This report has been made according to the SCARE criteria [4].

## 2 Presentation of case

**Fig. 1:**
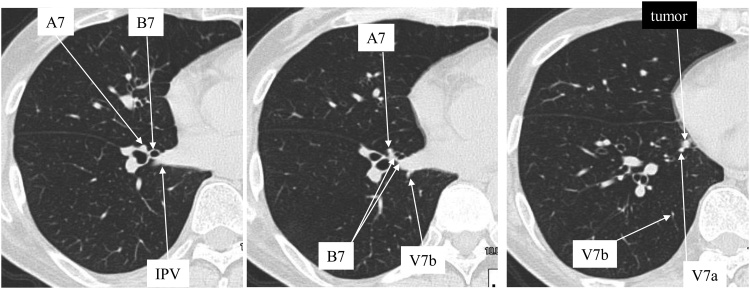
Preoperative computed tomography. A7 and B7 branch from the basal artery and basal bronchus, respectively, to run on the ventral side of the IPV. Therefore, the entire S7 is located ventral to the IPV. A 6-mm nodule can be seen in S7 adjacent to V7a. A thin V7b drains into V8-10.

**Fig. 2:**
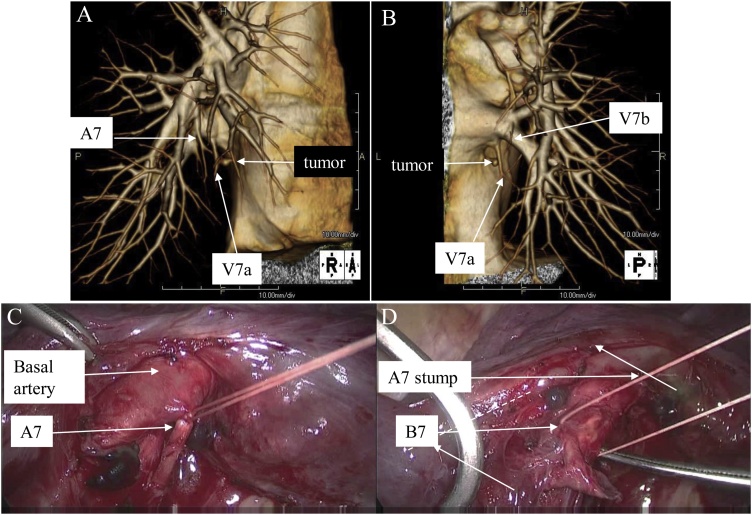
Three-dimensional CT and intraoperative findings (A) Three-dimensional CT in the right anterior oblique view showing the location of the tumor and the pulmonary vessels. (B) Three-dimensional CT in the posterior view shows V7b (marking the intersegmental plane between S7 and S10). (C)A7 branching from the basal artery was ligated. (D) B7 was isolated from behind A7.

A 56-year-old man was referred to our thoracic surgery department for resection of a pulmonary metastasis of rectal cancer. Low anterior resection for rectal cancer had been performed 17 months previously. Four months before presentation, follow-up computed tomography (CT) showed a 4 mm pulmonary metastasis in the right S7 and the lesion increased to 6 mm in diameter over the next 3 months. There was no evidence of other metastatic lesions, including lymph nodes metastasis or bone metastasis, on CT of the chest, abdomen, and pelvis. Laboratory tests were normal, including carcinoembryonic antigen. We selected S7 segmentectomy to obtain secure surgical margins. Preoperative CT showed that the entire S7 was located ventral to the inferior pulmonary vein (IPV), with A7 and B7 respectively branching from the basal segmental artery and bronchus on the ventral side of the IPV to enter S7 (Fig. 1, Fig. 2A). Under general anesthesia with differential lung ventilation, the patient was placed in the left lateral decubitus position. A 15 mm trocar was inserted into the right 8th intercostal space (ICS) on the midclavicular line for thoracoscopy. Next, a 4 cm mini-thoracotomy incision was made in the 5th ICS on the anterior axillary line for the operator and a 3 cm mini-thoracotomy incision was made in the 6th ICS dorsal to the scapula for the assistant. First, the pulmonary ligament was cut, after which V7a (first branch of the IPV) was ligated and cut. Next, the lung parenchyma was transected between the middle and lower lobes, and the interlobar pulmonary artery was identified. The A7 artery branching to the mediastinal side was isolated, ligated, and cut (Fig. 2C). Then B7 was identified between the IPV and A7 artery at a location dorsal to the A7 artery (Fig. 2D). To find the intersegmental plane, a bronchoscope was inserted through a double-lumen tube and wedged into the B7 bronchus. Selective delivery of oxygen into the B7 bronchus via the instrument channel of the bronchoscope allowed the part of the lung for resection (inflated) to be distinguished from that for preservation (collapsed). After the demarcation line was marked by electrocautery, the B7 bronchus was dissected by using a stapler. Then the stump of the B7 bronchus was ligated with 2-0 silk and elevated, followed by division of the intersegmental plane on the central side along the V8 vein with electrocautery. Subsequently, the V7b vein (marking the intersegmental plane between S7 and S10) was exposed and the plane was divided on the central side between S7 and S10 with electrocautery. The peripheral part of intersegmental plane was divided with a stapler. Finally, the air leak was closed by a continuous over-and-over suture of 4-0 PDSII (Ethicon, Inc., Somerville, NJ). To prevent further air leaks, polyglycolic acid sheets (Neoveil®, Gunze Ltd., Kyoto, Japan) were applied with fibrin glue (Beriplast P Combi-Set®, CSL Behring Pharma, Tokyo, Japan). The operating time was 175 min and blood loss was 10 ml. There were no complications of surgery and the patient was discharged on postoperative day 8. Histologically, the tumor was confirmed to be a pulmonary metastasis of rectal cancer with negative surgical margins were. So far, there has been no recurrence for 42 months after pulmonary metastasectomy.

## 3 Discussion

Although S7 segmentectomy is an uncommon procedure, it can be performed thoracoscopically if the anatomical relations are carefully assessed before surgery.

Only a few cases of thoracoscopic isolated S7 segmentectomy have been reported [2,3]. The reasons for S7 segmentectomy being rare include the small volume of this segment and its anatomical relations. Based on three-dimensional lung volume image analysis, S7 has the smallest volume of all lung segments and accounts for only 2.7% of the entire lung volume. In addition, theoretical analysis has estimated that there is only about a 20% possibility of achieving segmentectomy with a surgical margin of 2 cm for a 1 cm tumor in S7 [1]. Anatomical variation also makes it difficult to perform S7 segmentectomy. Chujo et al. [5] and Nagashima et al. [6] independently reported on the anatomical variations of S7. Chujo et al. classified the patterns of B7 and A7 branching into 5 types, while Nagashima et al. classified the B7 branching patterns into 4 types. Thee branching patterns can be broadly classified into three types, in which B7 (A7) runs ventral to the IPV (B7a type), B7 (A7) runs dorsal to the IPV (B7b type), or two branches of B7 (A7) run both dorsal and ventral to the IPV (B7ab type). In both studies, the first type was the most common (64% and 75%, respectively), and the present case also had this type of S7. With this type, B7 branches from the basal bronchus and A7 branches from the basal artery to run ventral to the IPV, which means that S7 is located entirely ventral to the IPV. When approached from the interlobar fissure during surgery, A7 is identified first, B7 is found behind A7, and the IPV is seen posterior to B7. Since the intersegmental plane is located ventral to the IPV, segmentectomy can be completed via the interlobar fissure approach, which means that thoracoscopic S7 segmentectomy with the operator positioned on the ventral side of the patient is a feasible procedure.

Regarding the other anatomical types, it is considered impossible to perform isolated segmentectomy when S7 surrounds the IPV because the intersegmental plane is divided by the IPV. Instead, sub-segmentectomy (S7a or S7b) is indicated for this type, as reported by Shimizu et al. [3].

There have been no reports of isolated segmentectomy when the entire S7 exists dorsal to the IPV. Based on the findings in the present case, we suggest that A7 branching from the basal artery and descending on the dorsal side of the basal bronchus may not be divided via the interlobar fissure approach with the operator positioned on the ventral side of the patient. Instead, a dorsal approach would be necessary to divide A7 and B7. Although thoracoscopic segmentectomy is considered feasible for this anatomical type of S7, it would be difficult using our method with the operator on the ventral side of the patient, and the thoracoscopic approach to this type of S7 may require some ingenuity.

## 4 Conclusion

Thoracoscopic S7 segmentectomy is feasible, depending on the anatomical features of S7.

If the local anatomy can be fully investigated before surgery, thoracoscopic isolated S7 segmentectomy may be a reasonable treatment option.

## Conflicts of interest

None.

## Sources of funding

This research did not receive any specific grant from funding agencies in the public, commercial, or not-for-profit sectors.

## Ethical approval

This is case report is exempt for ethical approval in our institute.

## Consent

Patient was advised that their clinical data could be used for various studies and comprehensive informed consent was obtained on that basis.

## Author contribution

NK and HT drafted the manuscript. NK, SS, and MT carried out this work. HT and AT supervised the project. All authors discussed the results and commented on the

manuscript.

## Registration of research studies

Not applicable.

## Guarantor

Naoya Kawakita.

## Provenance and peer review

Not commissioned, externally peer reviewed.

## References

[1] Ueda K., Tanaka T., Hayashi M. (2012). What proportion of lung cancers can be operated by segmentectomy? A computed-tomography-based simulation. Eur. J. Cardiothorac. Surg..

[2] Leshnower B.G., Miller D.L., Fernandez F.G. (2010). Video-assisted thoracoscopic surgery segmentectomy: a safe and effective procedure. Ann. Thorac. Surg..

[3] Shimizu K., Nagashima T., Yajima T. (2017). Thoracoscopic medial-basal segment segmentectomy. Ann. Thorac. Surg..

[4] Agha R.A., Fowler A.J., Saeta A. (2016). The SCARE statement: consensus-based surgical case report guidelines. Int. J. Surg..

[5] Chujo M., Anami K. (2014). Branching patterns of segmental bronchi and arteries in the medial basal segment. J. Bronchology Interv. Pulmonol..

[6] Nagashima T., Shimizu K., Ohtaki Y. (2017). Analysis of variation in bronchovascular pattern of the right middle and lower lobes of the lung using three-dimensional CT angiography and bronchography. Gen. Thorac. Cardiovasc. Surg..

